# Management Strategies and Patient Selection After a Hospital Funding Reform for Prostate Cancer Surgery in Canada

**DOI:** 10.1001/jamanetworkopen.2019.10505

**Published:** 2019-08-30

**Authors:** Marian S. Wettstein, Karen S. Palmer, Girish S. Kulkarni, J. Michael Paterson, Vicki Ling, Lauren Lapointe-Shaw, Alvin H. Li, Adalsteinn Brown, Monica Taljaard, Noah Ivers

**Affiliations:** 1 Division of Urology, Department of Surgery, Princess Margaret Cancer Centre, University Health Network, University of Toronto, Toronto, Ontario, Canada; 2 Institute for Health Policy, Management and Evaluation, University of Toronto, Toronto, Ontario, Canada; 3 Faculty of Health Sciences, Simon Fraser University, Burnaby, British Columbia, Canada; 4 Women’s College Research Institute, Women’s College Hospital, Toronto, Ontario, Canada; 5 ICES, Toronto, Ontario, Canada; 6 Department of Medicine, University of Toronto, Toronto, Ontario, Canada; 7 Ottawa Hospital Research Institute, Ottawa, Ontario, Canada; 8 Dalla Lana School of Public Health, University of Toronto, Toronto, Ontario, Canada; 9 Department of Obstetrics and Gynecology, University of Toronto, Toronto, Ontario, Canada; 10 School of Epidemiology and Public Health, University of Ottawa, Ottawa, Ontario, Canada; 11 Department of Family and Community Medicine, University of Toronto, Toronto, Ontario, Canada

## Abstract

**Importance:**

Hospital funding reforms for prostate cancer surgery may have altered management of localized prostate cancer in the province of Ontario, Canada.

**Objective:**

To determine whether changes in hospital funding policy aimed at improving health care quality and value were associated with changes in the management of localized prostate cancer or the characteristics of patients receiving radical prostatectomy (RP) for localized prostate cancer.

**Design, Setting, and Participants:**

This population-based, interrupted time series study used linked population-based administrative data regarding adults in Ontario with incidental localized prostate cancer and those who underwent RP for localized prostate cancer. Patients who underwent RP were compared with patients who underwent surgical procedures for localized renal cell carcinoma, which was not included in the policy change but was subjected to similar secular trends and potential confounders. Monthly outcomes were analyzed using interventional autoregressive integrated moving average models. Data were collected from January 2011 to November 2017 and analyzed in January 2019.

**Exposures:**

Funding policy change in April 2015 from flexible block funding for all hospital-based care to prespecified payment amounts per procedure for treatment of localized prostate cancer, coupled with the dissemination of a diagnosis-specific handbook outlining best practices.

**Main Outcomes and Measures:**

Initial management (RP vs radiation therapy vs active surveillance) and tumor risk profiles per management strategy among incident cases of localized prostate cancer. Additional outcomes were case volume, mean length of stay, proportion of patients returning to hospital or emergency department within 30 days, proportion of patients older than 65 years, mean Charlson Comorbidity Index, and proportion of minimally invasive surgical procedures among patients undergoing RP for localized prostate cancer.

**Results:**

A total of 33 128 patients with incident localized prostate cancer (median [interquartile range (IQR)] age, 67 [61-73] years; median [IQR] cases per monthly observation interval, 466 [420-516]), 17 159 patients who received radical prostatectomy (median [IQR] age, 63 [58-68] years; median [IQR] cases per monthly observation interval, 209 [183-225]), and 5762 individuals who underwent surgery for renal cell carcinoma (median [IQR] age, 62 [53-70] years; median [IQR] cases per monthly observation interval, 71 [61-77]) were identified. By the end of the observation period, radical prostatectomy and radiation therapy were used in comparable proportions (30.3% and 28.9%, respectively) and included only a small fraction of low-risk patients (6.4% and 2.9%, respectively). No statistically significant association of the funding policy change with most outcomes was found.

**Conclusions and Relevance:**

The implementation of funding reform for hospitals offering RP was not associated with changes in the management of localized prostate cancer, although it may have encouraged more appropriate selection of patients for RP. Mostly preexisting trends toward guideline-conforming practice were observed. Co-occurring policy changes and/or guideline revisions may have weakened signals from the policy change.

## Key Points

**Question:**

Was a change in hospital funding policy for radical prostatectomy associated with changes in management of localized prostate cancer in the province of Ontario, Canada?

**Findings:**

In this population-based interrupted time series study, which included 33 128 patients with incident localized prostate cancer and 17 159 patients treated with radical prostatectomy, no statistically significant association of the change in hospital funding policy with most outcomes was found. However, potential improvement in appropriate patient selection for prostate cancer surgery was observed.

**Meaning:**

The implementation of a hospital funding policy change aimed at improving health care quality and value was not associated with management of localized prostate cancer.

## Teaser

This population-based, interrupted time series study evaluates whether changes in hospital funding policy in Ontario, Canada, aimed at improving health care quality and value were associated with changes in the management of localized prostate cancer or the characteristics of patients receiving radical prostatectomy for localized prostate cancer.

## Introduction

Prostate cancer (PC) is the most commonly diagnosed malignant neoplasm among North American men and a leading cause of male cancer deaths.^1^ Guidelines emphasize the careful selection of appropriate management strategies for localized PC, ie, radical prostatectomy (RP) vs radiation therapy vs active surveillance, to best balance the risks of cancer-associated mortality with the risks of treatment-associated morbidity.^2,3^

On May 3, 2010, the government of Ontario, Canada, issued a press release announcing its intention to move forward with hospital funding reforms aimed at improving health care quality and value.^4^ These policy reforms were intended to make the health care system more patient-centered, smarter in its use of limited resources, more sustainable, and more focused on quality.^5^

A key element of this policy transformation was quality-based procedures (QBPs), a reform designed to eventually shift a portion of hospital funding (originally envisioned at approximately 30%^6^) from annual global budgets to patient-focused funding. Under QBPs, hospitals are reimbursed for certain procedures and diagnoses according to a prespecified (prospective) payment multiplied by a predetermined annual volume (ie, price × volume) with quality intended to be accounted for through discretionary adherence to best clinical practices outlined in handbooks specific to each QBP.^7^ The provincial government determined prespecified reimbursement rates for each QBP through an extensive case-costing exercise; annual volumes are based on previous year volumes. There are no financial penalties for noncompliance with best practices described in the handbooks, as the handbooks were intended to have only an educational or exhortational effect. Physician payment is not included in the QBP procedure or diagnosis fee; physicians continue to bill the government separately for their services. New QBPs, identified annually, were phased in each fiscal year starting April 1, 2012, as part of a multiyear implementation plan.^4^ The clinical handbook for RP was first published January 12, 2015,^8,9^ and QBP funding for RP began April 1, 2015.

We hypothesized that this funding policy change might be associated with changes in the management of localized PC, including changes in patient selection for RP vs other management strategies. It is also plausible that the fixed annual price and volume might incentivize adverse risk selection, ie, cherry-picking, with only patients expected to have a short length of stay selected for RP (to maximize revenue for the hospital) and those expected to have a longer length of stay being offered radiation or active surveillance rather than surgery.^10^ Hence, we studied the associations of this hospital funding reform with the following: (1) management of localized PC (RP vs radiation vs active surveillance) and (2) patient characteristics of males undergoing RP for localized PC. We compared patients who underwent RP for localized PC with a urologic comparator cohort not funded through QBP but affected by similar secular trends and potential confounders (surgery for localized renal cell carcinoma [RCC]).

## Methods

### Design

We designed a retrospective, population-based interrupted time series (ITS) study using linked health administrative data held and analyzed at ICES (Toronto, Ontario, Canada). We conducted interventional autoregressive integrated moving average (ARIMA) analyses in January 2019 to evaluate changes associated with the implementation of QBP funding. Interventional ARIMA, originally developed in econometrics, is a very robust method that, in comparison with other analytical approaches (eg, a simple before-vs-after comparison) captures any systematic time series patterns, such as nonstationarity, autocorrelation, and seasonality.^11^ Additional multivariable adjustment is not necessary in most situations as the method further accounts for measured and unmeasured confounders that gradually change over time (eg, tumor risk profile at surgery).^12^ Ethics approval was waived by the Sunnybrook Health Sciences Centre research ethics board because ICES is a prescribed entity that does not require informed consent, and reporting was in accordance with an ITS-specific extension of the Strengthening the Reporting of Observational Studies in Epidemiology (STROBE) reporting guideline.^13,14^

### Denominator of Time Series

We created 3 distinct time series that were aggregated in monthly intervals. Series 1 consisted of men diagnosed with localized PC from January 2011 to October 2016 (indexed at date of diagnosis). Series 2 and series 3 consisted of men who underwent RP for localized PC and surgery for localized RCC, respectively, from January 2011 to November 2017 (indexed at date of discharge). We excluded patients younger than 18 years or older than 105 years, individuals not covered by the Ontario Health Insurance Plan through the study period, and those with any other cancer diagnosis before or after the index date.

### Data Sources

Series 1 was identified by the Ontario Cancer Registry, which captures information regarding all incident cancer cases in Ontario, including relevant staging information at the time of diagnosis.^15^ Series 2 and series 3 were identified through the Canadian Institute for Health Information Discharge Abstract Database, which captures administrative, clinical, and demographic information on all hospital discharges, with the index date being the date of discharge. To ascertain the time series outcomes, data for patients identified through these repositories were linked to other data sources, including the Ontario Health Insurance Plan Claims History Database, Canadian Institute for Health Information National Ambulatory Care Reporting System database, Canadian Institute for Health Information Same Day Surgery Database, and the Registered Person Database. The definitions we used for identifying the time series and ascertaining the associated outcomes are listed in eTable 1 in the Supplement.

### Management of Patients Diagnosed With Localized PC

Patients in series 1 were observed for 14 months for the first occurrence of RP, radiation therapy, or prostate biopsy (a marker for active surveillance) to determine the initial management strategy. We chose this observation window to capture men in an active surveillance scheme who could not receive their rebiopsy within the recommended interval of 6 to 12 months^16^ owing to either patient indecision or availability, health system wait times, or other factors. In addition, we determined tumor characteristics, mean prostate-specific antigen at time of diagnosis, clinical T stage (ie, cT1, cT2, or ≥cT3), and Gleason score at time of diagnosis (ie, ≤6, 7, or ≥8) to derive the D’Amico risk group classification (ie, low, intermediate, or high) for each individual.^17^ The proportions of D’Amico low-risk patients among the initial management strategies were then analyzed as additional time series.

### Characteristics of Patients Undergoing RP for Localized PC or Surgery for Localized RCC

Series 2 and series 3 were limited to men who underwent surgery in QBP-funded hospitals. To further improve comparability between the cohorts, we excluded patients with RCC who underwent repair of the vena cava and/or a thoracoabdominal procedure, suggestive of advanced disease. In both series, we ascertained the following outcomes: volume, mean length of stay (ie, time from admission to discharge in hours), proportion of patients returning to hospital or emergency department (ED) within 30 days, proportion of patients older than 65 years at date of discharge, mean Charlson Comorbidity Index (CCI) (based on a lookback window of 3 years from date of discharge), and proportion of patients who received a minimally invasive approach (ie, robot-assisted or conventional laparoscopic approach vs open surgery). To determine the proportion of patients who returned to the hospital or ED within 30 days, we excluded patients who died during hospitalization, were transferred to another hospital or ambulatory care center, or who signed out against medical advice. We further evaluated series 2 and series 3 among the subgroups of high- and low-volume hospitals to explore whether potential associations of the policy change with the outcomes were conditional on case load. Dichotomization occurred at an annual mean RP volume of 100 cases per year.

### Statistical Analysis

We fit ARIMA models to the time series before the interruption point and compared the data point observed after the interruption point with those predicted by the model’s 95% CIs. Model selection was guided by inspection of the autocorrelation and partial autocorrelation function of the detrended series. The presence of white noise was verified by examining the autocorrelations at various lags with the Ljung-Box χ^2^ statistic. The final models were empirically chosen based on the Bayesian information criterion. Effect estimates were presented as the difference between the mean observed and the mean predicted value (Δ_predicted−observed_), both calculated across the 12 months immediately following the interruption point. We used the Wald test to investigate the hypothesis that the introduction of QBP led to a statistically significant change in the defined outcomes. The intervention was assumed to act as a step function with a 0-order response.^18^

We chose April 1, 2015 (start of Ontario government’s fiscal year), as the interruption point because the Ontario Ministry of Health and Long-Term Care initiated the QBP funding for RP on that date. Although the original press release in May 2010 announced the Ontario government’s intent to move toward a patient-based funding model, no candidate QBP procedures or diagnoses were announced then. Similarly, although the handbook for RP was first published online in January 2015, our prior research suggested that the extent to which hospitals were aware of this is largely unknown. Therefore, the date of the actual funding change at the start of the fiscal year was the most reliable interruption point.^6,7^ Data aggregation was performed in SAS version 9.4 (SAS Institute), and ARIMA modeling was implemented in R 3.4.4 (R Project for Statistical Computing; packages: astsa^19^ and TSA^20^). Statistical significance was assessed at *P* < .05, and all tests were 2-tailed.

## Results

Derivation of each time series is visualized in eFigure 1 in the Supplement. We identified 33 128 men with a primary diagnosis of localized PC (series 1; median [interquartile range (IQR)] cases per monthly observation interval, 466 [420-516]), 17 159 patients who received RP (series 2; median [IQR] cases per monthly observation interval, 209 [183-225]), and 5762 individuals who underwent surgery for RCC (series 3; median [IQR] cases per monthly observation interval, 71 [61-77]). The Table presents the composition (monthly intervals before and after policy change, median number of patients per monthly interval) and patient characteristics of each series. Patients in series 1 were older than patients in series 2 and series 3 (median [IQR] age, 67 [61-73] years vs 63 [58-68] years and 62 [53-70] years, respectively). Rurality as well as comorbidity (continuously measured by the CCI) among the cohorts were comparable. Higher socioeconomic status was more common in all 3 series, although the overrepresentation was considerably more pronounced among patients who received RP.

**Table.** Characteristics of the 3 Time Series

Characteristic	No. (%)		
	Series 1, Localized Prostate Cancer	Series 2, Radical Prostatectomy	Series 3, Surgery for RCC
No.	33 128	17 159	5762
Intervals before policy change, No.	51	51	51
Intervals after policy change, No.	19	32	32
Events per interval, median (IQR), No.	466 (420-516)	209 (183-225)	71 (61-77)
Age, median (IQR), y^a^	67 (61-73)	63 (58-68)	62 (53-70)
SES quintile^b^			
First	4888 (14.8)	2249 (13.1)	955 (16.6)
Second	6024 (18.2)	3001 (17.5)	1085 (18.8)
Third	6554 (19.8)	3357 (19.6)	1154 (20.0)
Fourth	7324 (22.1)	3942 (23.0)	1331 (23.1)
Fifth	8237 (24.9)	4578 (26.7)	1207 (20.9)
Missing	101 (0.3)	32 (0.2)	30 (0.5)
Rurality	4743 (14.3)	2452 (14.3)	870 (15.1)
CCI, median (IQR)	0 (0-0)	0 (0-0)	0 (0-0)
PSA, median (IQR), ng/mL^a^	7.4 (5.4-11.3)	NA	NA
Missing, No. (%)	4904 (14.8)	NA	NA
T stage^a^			
cT1	18 262 (55.1)	NA	NA
cT2	13 267 (40.0)	NA	NA
≥cT3	1375 (4.2)	NA	NA
Missing	224 (0.7)	NA	NA
Gleason score^a^			
≤6	10 871 (32.8)	NA	NA
7	15 570 (47.0)	NA	NA
≥8	5918 (17.9)	NA	NA
Missing	769 (2.3)	NA	NA
D’Amico risk group^a^			
Low	5776 (17.4)	NA	NA
Intermediate	11 339 (34.2)	NA	NA
High	10 681 (32.2)	NA	NA
Missing	5332 (16.1)	NA	NA
Surgical approach			
Open	NA	12 271 (71.5)	2615 (45.4)
Robot-assisted	NA	3801 (22.2)	157 (2.7)
Laparoscopic	NA	1087 (6.3)	2990 (51.9)
Teaching institution	NA	7503 (43.7)	2767 (48.0)
High-volume institution	NA	6810 (39.7)	1915 (33.2)

Abbreviations: CCI, Charlson Comorbidity Index; IQR, interquartile range; NA, not applicable; PSA, prostate-specific antigen; RCC, renal cell carcinoma; SES, socioeconomic status.

SI conversion factor: To convert PSA to micrograms per liter, multiply by 1.0.

^a^ At diagnosis (series 1) or date of discharge (series 2 and 3).

^b^ Postal code–based income quintiles; first denotes lowest quintile and fifth, highest.

### Management of Patients Diagnosed With Localized PC

**Figure 1.**
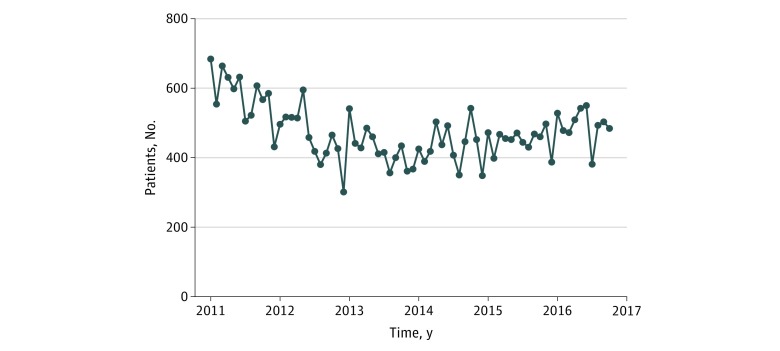
Monthly Incidence of Localized Prostate Cancer in Ontario, Canada, From January 2011 to October 2016

**Figure 2.**
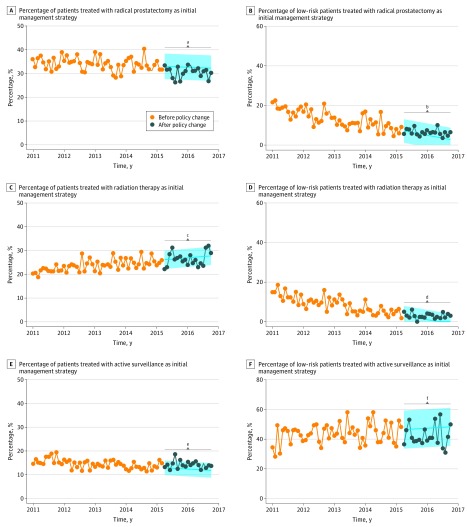
Initial Management of Localized Prostate Cancer Among 33 128 Patients and Proportion of Low-risk Patients in Each Management Strategy The shaded areas represent the predicted 95% CIs, and the *P* values are based on a step function with a 0-order response. ^a^*P* = .004. ^b^*P* = .56. ^c^*P* = .53. ^d^*P* = .67. ^e^*P* = .73. ^f^*P* = .42.

Among patients diagnosed with primary localized PC, the median (IQR) prostate-specific antigen value was 7.4 (5.4-11.3) ng/mL (to convert to micrograms per liter, multiply by 1.0). Most patients were diagnosed at a clinical T1 stage (18 252 [55.1%]) and at a Gleason score of 7 (15 570 [47.0%]). Owing to missing values (prostate-specific antigen, 4904 [14.8%]; T stage, 224 [0.7%]; and Gleason score, 769 [2.3%]), a D’Amico risk group could be assigned to only 5332 patients (83.9%) in series 1. Figure 1 displays the monthly incidence of localized PC in Ontario from January 2011 to October 2016. After a steep decrease, ending at the end of 2012, we observed a slight but stable increase in monthly diagnoses. The results of the ARIMA analysis investigating the proportion of each initial management strategy and the proportion of low-risk patients among each strategy are presented in Figure 2. Step functions and model specifications appear in eFigure 6A in the Supplement.

The proportion of patients who underwent RP as the initial management strategy showed a slight downward trend before the funding policy change, which became more pronounced after the policy change (Δ_predicted−observed_ = −2.0%; *P* = .004) (Figure 2A). Conversely, the proportion receiving radiation therapy as the initial management strategy demonstrated an upward trend that was not associated with the policy change (Δ_predicted−observed_ = −0.5%; *P* = .53) (Figure 2C). We observed a slight decrease in the proportion of patients receiving active surveillance as the initial management strategy before the policy change, which attenuated after the policy change without reaching statistical significance (Δ_predicted−observed_ = 1.8%; *P* = .73) (Figure 2E). The fraction of low-risk patients among the definitive treatment strategies (RP and radiation therapy) showed a stable downward trend that reached a plateau below 10%; it was not associated with the policy change (RP: Δ_predicted−observed_ = 0.8%; *P* = .56; radiation therapy: Δ_predicted−observed_ = 1.4%; *P* = .42) (Figure 2B and D). Among low-risk patients following an active surveillance approach, we observed high fluctuations and an upward trend before the policy change, with a possible attenuation afterward (Δ_predicted−observed_ = −5.4%; *P* = .42) (Figure 2F). By the end of the observation period (October 2016), RP and radiation therapy were used in comparable proportions (30.3% and 28.9%, respectively) and included only a very small fraction of low-risk patients (6.4% and 2.9%, respectively).

### Characteristics of Patients Undergoing RP for Localized PC and Surgery for Localized RCC

**Figure 3.**
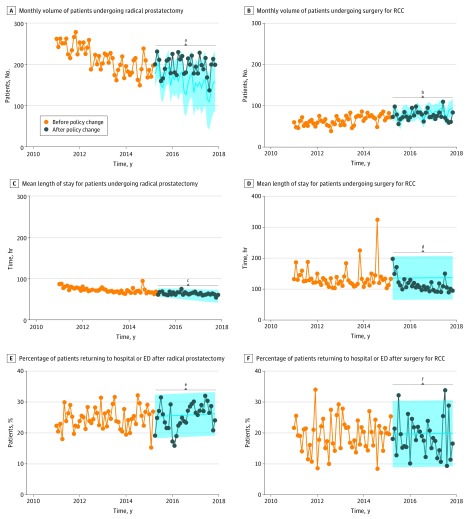
Monthly Volume, Mean Length of Stay, and Proportion of Patients Returning to the Hospital or Emergency Department (ED) Among 17 159 Patients Undergoing Radical Prostatectomy and 5762 Patients Undergoing Surgery for Renal Cell Carcinoma (RCC) The shaded areas represent the predicted 95% CIs, and the *P* values are based on a step function with a 0-order response. ^a^*P* = .06. ^b^*P* = .93. ^c^*P* = .28. ^d^*P* = .80. ^e^*P* = .74. ^f^*P* = .61.

**Figure 4.**
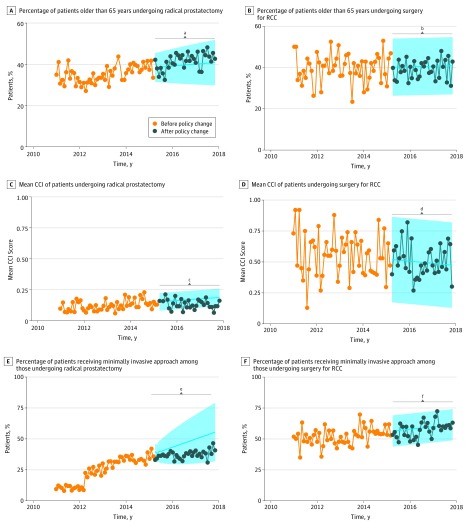
Proportion of Patients Older Than 65 Years, Mean Charlson Comorbidity Index (CCI) Score, and Proportion of Patients Receiving Minimally Invasive Approach Among 17 159 Patients Undergoing Radical Prostatectomy and 5762 Patients Undergoing Surgery for Renal Cell Carcinoma (RCC) The shaded areas represent the predicted 95% CIs, and the *P* values are based on a step function with a 0-order response. ^a^*P* = .82. ^b^*P* = .75. ^c^*P* = .73. ^d^*P* = .15. ^e^*P* = .19. ^f^*P* = .80.

Figure 3 and Figure 4 show the ARIMA analysis of the characteristics of patients undergoing RP and surgery for RCC. Step functions and model specifications appear in eFigure 6B and C in the Supplement. Heterogeneity in the shape of the confidence bands is explained by different model specifications (eg, seasonal vs nonseasonal ARIMA models). The monthly volume of RP showed a strong downward trend before the policy change that plateaued after the policy change (Δ_predicted−observed_ = 21.8 cases; *P* = .06) (Figure 3A). The monthly volume of surgery for RCC steadily increased regardless of the policy change (Δ_predicted−observed_ = −2.0 cases; *P* = .93) (Figure 3B). The mean length of stay decreased in both cohorts, not associated with the policy change (RP: Δ_predicted−observed_ = −0.9 hours; *P* = .28; surgery for RCC: Δ_predicted−observed_ = −5.2 hours; *P* = .80) (Figure 3C and D), although the RCC data exhibited higher variability, influenced by extreme values. The proportion of patients who returned to the hospital or ED within 30 days after the procedure demonstrated substantial variability in both series, not associated with the funding policy change (RP: Δ_predicted−observed_ = −2.4%; *P* = .74; surgery for RCC: Δ_predicted−observed_ = −0.2%; *P* = .61) (Figure 3E and F).

Among patients who underwent RP, the fraction of individuals older than 65 years initially decreased, reached a nadir in 2012, and then steadily increased (Δ_predicted−observed_ = 0.2%; *P* = .82) (Figure 4A). In the series of patients older than 65 years who underwent surgery for RCC, we observed a higher variability but could not detect a trend (Δ_predicted−observed_ = −1.7%; *P* = .75) (Figure 4B). The mean CCI in the cohort of patients undergoing RP demonstrated an upward trend before the QBP policy change that attenuated after the policy change without reaching statistical significance (Δ_predicted−observed_ = −0.01; *P* = .73) (Figure 4C). For RCC, the mean CCI showed greater variability and a downward trend not associated with the policy change (Δ_predicted−observed_ = 0.04; *P* = .15) (Figure 4D). We observed no trend prior to 2012 in the proportion of minimally invasive RPs and then a high step followed by a steep upward trend, which attenuated after the policy change (Δ_predicted−observed_ = −5.1%; *P* = .19) (Figure 4E). The fraction of minimally invasive surgical procedures for RCC showed a stable increase not associated with the policy change (Δ_predicted−observed_ = −3.0%; *P* = .80) (Figure 4F).

Overall, 5 of the 60 relevant hospitals in Ontario (8.3%) performed an average of more than 100 RPs per year and were considered high-volume PC hospitals. Of patients who underwent RP or surgery for RCC, 6810 (39.7%) and 1915 (33.2%), respectively, had their surgical procedures in a high-volume hospital. The ITS analyses stratified by volume are illustrated in eFigures 2-5 in the Supplement (qualitative summary appears in eTable 2 in the Supplement, step functions and model specifications in eFigure 6D-G in the Supplement). For both procedures, we observed that high-volume hospitals showed much less variability in monthly case volumes compared with low-volume hospitals. High-volume hospitals also showed an upward trend in the proportion of patients returning to the hospital or ED, but this was not associated with the funding policy change. Among the patients receiving RP, we further detected that the subgroup of individuals receiving the procedure in high-volume hospitals had a less pronounced upward trend in mean CCI before the funding policy change but a much steeper increase in the proportion of minimally invasive procedures.

## Discussion

In this retrospective, population-wide ITS study, we used ARIMA modeling to evaluate whether the implementation of a new funding model for hospitals offering RP led to changes in the management of localized PC and/or the characteristics of patients undergoing RP for localized PC. We found mostly favorable preexisting trends that were unaffected by the policy change.

By the end of the observation period (October 2016), the definitive management strategies (RP and radiation therapy) were used in comparable proportions (30.3% and 28.9%, respectively) and included only a very small fraction of low-risk patients (6.4% and 2.9%, respectively). Our findings demonstrate that localized PC is treated in Ontario in accordance with guidelines and that the funding change was not negatively associated with management of these patients.

Among patients receiving RP for localized PC, the funding change was not associated with either length of stay or the proportion of patients returning to the hospital or ED. However, substantially fewer patients received RP as the initial management strategy after the policy change, while the mean monthly RP volume simultaneously increased, suggesting improved patient selection and/or that the funding reform helped hospitals address unmet needs for the procedure. The upward trend (unassociated with the funding policy change) in the proportion of patients older than 65 years alongside an attenuation in the increase in mean CCI suggests that urologists were increasingly (and appropriately) selecting patients for RP based on a biological age concept (life expectancy >10 years) rather than on chronological age alone and that the policy change did not seem to inflate comorbidity coding. However, the interpretation of any potential changes associated with the funding policy on the fraction of minimally invasive procedures is limited as the appearance of the time series is mainly driven by 3 co-occurring phenomena: (1) the limited use of conventional laparoscopy in prostate cancer surgery, (2) the sudden appearance and steep increase in robot-assisted procedures in 2012, and (3) the nonexistent public funding for robot-assisted surgical procedures.

### Limitations

To our knowledge, this is the first study that used an ITS design to comprehensively investigate the association of a price × volume funding reform for PC with outcomes. All findings should be interpreted in the complex context of national and international PC screening recommendations against routine PC screening, which likely contributed to Ontario’s steep decrease in new PC diagnoses that reached its nadir at the end of 2013 and slowly increased afterward. The statistical conclusion of an interventional ARIMA is potentially subjective to model specification, selection of the interruption point, and definition of the response function. The step function with a 0-order response was chosen because of its relative simplicity with limited underlying assumptions. We provide time series plots displaying both the observed values (before and after the policy change) and the predicted CIs to allow for interpretation of the analysis apart from the statistical hypothesis test.

From a clinical perspective, our work provides evidence of satisfactory PC treatment trends that were not disrupted by implementation of the QBP funding and dissemination of best-practice handbooks. From a policy perspective, our study demonstrates the challenge of evaluating the impact of an intervention in a complex environment with numerous co-occurring policy changes (eg, centralization of care and remuneration changes at the physician level) and guideline revisions that might mask or confound potential effects. Our prior work describes QBPs in detail and analyzes their implementation, demonstrating the challenge of detecting a policy signal across several QBP-funded diseases and procedures, in a setting with considerable co-occurring noise (A.H.L., unpublished data, January 2019) where policy implementation was challenging.^6,7^ We narrowed this study to include a single disease (localized PC), and we further compared competing management strategies (RP vs radiation therapy vs active surveillance), performed subgroup analyses according to hospital volumes, and assessed a urologic comparator cohort not funded through QBP.^21^ Nevertheless, disentangling the changes associated with this policy from co-occurring trends was challenging. Under consideration of previous studies suggesting that guidelines and evidence have relatively weak associations with care compared with other sources of guidance,^22,23,24^ the apparently small association of funding reform coupled with best practice handbooks, in contrast with trends toward more appropriate care, deserves closer study. Future studies using mixed methods might be helpful to understand physician perception of the relative effect of funding model changes on their practice.

## Conclusions

Overall, the implementation of a price × volume reimbursement model for hospitals offering RP, along with discretionary adherence to best-practice clinical handbooks, did not appear to be associated with changes in the management of localized PC. We did not see evidence of cherry-picking for patients most likely to have a short length of stay to maximize hospitals’ financial gain. We observed mostly preexisting favorable trends, concordant with co-occurring guidelines aimed at maximizing detection of aggressive and potentially lethal disease while minimizing harms associated with unnecessary prostate biopsy and with the detection of clinically insignificant prostate cancer.^25^ The funding reform may have encouraged even more desirable patient selection for RP. However, assessment of potential positive and negative consequences of these policy changes is highly challenging in the presence of dominant underlying preexisting trends.

## Notes

Supplement. **eTable 1.** Definitions **eTable 2.** Qualitative Summary of Subgroup Analyses **eFigure 1.** Derivation of Time Series **eFigure 2.** Monthly Volume, Mean Length of Stay, and Proportion of Patients Returning to the Hospital or the Emergency Department Among the Subgroup of Patients Undergoing Radical Prostatectomy (N = 6810) and Surgery for Renal Cell Carcinoma (N = 1915) in High-Volume Hospitals **eFigure 3.** Proportion of Patients Older Than 65 Years, Mean Charlson Comorbidity Index, and Proportion of Patients Who Received Minimally Invasive Approach Among Subgroup of Patients Undergoing Radical Prostatectomy (N = 6810) and Surgery for Renal Cell Carcinoma (N = 1915) in High-Volume Hospitals **eFigure 4.** Monthly Volume, Mean Length of Stay, and Proportion of Patients Returning to the Hospital or the Emergency Department Among the Subgroup of Patients Undergoing Radical Prostatectomy (N = 10 349) and Surgery for Renal Cell Carcinoma (N = 3874) in Low-Volume Hospitals **eFigure 5.** Proportion of Patients Older Than 65 Years, Mean Charlson Comorbidity Index, and Proportion of Patients Who Received Minimally Invasive Approach Among Subgroup of Patients Undergoing Radical Prostatectomy (N = 10 349) and Surgery for Renal Cell Carcinoma (N = 3874) in Low-Volume Hospitals **eFigure 6.** Step Functions and Model Specifications
