# Supplementary material for: Management Strategies and Patient Selection After a Hospital Funding Reform for Prostate Cancer Surgery in Canada
Source: JAMA Netw Open. 2019 Aug 30;2(8):e1910505. doi: 10.1001/jamanetworkopen.2019.10505 (PMC6724173; doi:10.1001/jamanetworkopen.2019.10505)
Supplement: Supplement. — eTable 1. Definitions eTable 2. Qualitative Summary of Subgroup Analyses eFigure 1. Derivation of Time Series eFigure 2. Monthly Volume, Mean Length of Stay, and Proportion of Patients Returning to the Hospital or the Emergency Department Among the Subgroup of Patients Undergoing Radical Prostatectomy (N = 6810) and Surgery for Renal Cell Carcinoma (N = 1915) in High-Volume Hospitals eFigure 3. Proportion of Patients Older Than 65 Years, Mean Charlson Comorbidity Index, and Proportion of Patients Who Received Minimally Invasive Approach Among Subgroup of Patients Undergoing Radical Prostatectomy (N = 6810) and Surgery for Renal Cell Carcinoma (N = 1915) in High-Volume Hospitals eFigure 4. Monthly Volume, Mean Length of Stay, and Proportion of Patients Returning to the Hospital or the Emergency Department Among the Subgroup of Patients Undergoing Radical Prostatectomy (N = 10 349) and Surgery for Renal Cell Carcinoma (N = 3874) in Low-Volume Hospitals eFigure 5. Proportion of Patients Older Than 65 Years, Mean Charlson Comorbidity Index, and Proportion of Patients Who Received Minimally Invasive Approach Among Subgroup of Patients Undergoing Radical Prostatectomy (N = 10 349) and Surgery for Renal Cell Carcinoma (N = 3874) in Low-Volume Hospitals eFigure 6. Step Functions and Model Specifications [file jamanetwopen-2-e1910505-s001.pdf]

Supplementary Online Content

Wettstein MS, Palmer KS, Kulkarni GS, et al. Management strategies and patient selection after a hospital funding reform for prostate cancer surgery in Canada. *JAMA Netw Open*. 2019;2(8):e1910505. doi:10.1001/jamanetworkopen.2019.10505

eTable 1. Definitions
eTable 2. Qualitative Summary of Subgroup Analyses
eFigure 1. Derivation of Time Series
eFigure 2. Monthly Volume, Mean Length of Stay, and Proportion of Patients Returning to the Hospital or the Emergency Department Among the Subgroup of Patients Undergoing Radical Prostatectomy (N = 6810) and Surgery for Renal Cell Carcinoma (N = 1915) in High-Volume Hospitals
eFigure 3. Proportion of Patients Older Than 65 Years, Mean Charlson Comorbidity Index, and Proportion of Patients Who Received Minimally Invasive Approach Among Subgroup of Patients Undergoing Radical Prostatectomy (N = 6810) and Surgery for Renal Cell Carcinoma (N = 1915) in High-Volume Hospitals
eFigure 4. Monthly Volume, Mean Length of Stay, and Proportion of Patients Returning to the Hospital or the Emergency Department Among the Subgroup of Patients Undergoing Radical Prostatectomy (N = 10 349) and Surgery for Renal Cell Carcinoma (N = 3874) in Low-Volume Hospitals
eFigure 5. Proportion of Patients Older Than 65 Years, Mean Charlson Comorbidity Index, and Proportion of Patients Who Received Minimally Invasive Approach Among Subgroup of Patients Undergoing Radical Prostatectomy (N = 10 349) and Surgery for Renal Cell Carcinoma (N = 3874) in Low-Volume Hospitals
eFigure 6. Step Functions and Model Specifications

This supplementary material has been provided by the authors to give readers additional information about their work.

© 2019 Wettstein MS et al. *JAMA Network Open.*

eTable 1. Definitions

Concept	Codes and definitions	Data source
Series 1		
Patients diagnosed with localized prostate cancer	PSITE = C61.9<br><br>AND<br><br>CSMETSDX not 00 or 99<br><br>AND<br><br>Date of diagnosis between January 1, 2011 and October 31, 2016	Ontario Cancer Registry
Radical prostatectomy	INCDODE = 1.QT.91	Canadian Institute for Health Information Discharge Abstract Database / National Ambulatory Care Reporting System / Same Day Surgery
	Fee codes = S651, S653, S645 or S646	Ontario Health Insurance Plan
Radiation therapy	INCDODE = : 1.QT.26, 1.QT27 or 1.QT53	Canadian Institute for Health Information Discharge Abstract Database / National Ambulatory Care Reporting System / Same Day Surgery
	Fee codes = X310, X311, X312, X313, S640, X322, X323, X324, X325 or X334	Ontario Health Insurance Plan
Prostate biopsy	INCDODE = 2.QT.71	Canadian Institute for Health Information Discharge Abstract Database / National Ambulatory Care Reporting System / Same Day Surgery
	Fee codes = Z712, Z713, S644, E780	Ontario Health Insurance Plan
Death		Registered Person Database
Prostate-specific antigen value at diagnosis	CSSSF1	Ontario Cancer Registry
Gleason Score at diagnosis	CSSSF8	Ontario Cancer Registry
T stage at diagnosis	CSEXT	Ontario Cancer Registry

© 2019 Wettstein MS et al. *JAMA Network Open.*

Concept	Codes and definitions	Data source
Series 2 and Series 3		
Patients undergoing radical prostatectomy for localized prostate cancer	DX10CODE = C61<br><br>AND<br><br>INCODE = 1QT91<br><br>AND<br><br>Date of discharge between January 1, 2011 and November 30, 2017	Canadian Institute for Health Information Discharge Abstract Database
Radical prostatectomy	• "Robot-assisted" if 7.SF.14 present (INCODE)<br>• "Laparoscopic" if 1.QT.91.DA is present in the absence of 7.SF.14 (INCODE)<br>• Otherwise "Open"	Canadian Institute for Health Information Discharge Abstract Database
Patients undergoing surgery for renal cell carcinoma	DX10CODE = C64 but not 80006 or 80106<br><br>AND<br><br>INCODE = 1P87, 1PC91 (except 1PC91QF), 1PD89 (except 1PD89QF)<br><br>AND<br><br>Date of discharge between January 1, 2011 and November 30, 2017	Canadian Institute for Health Information Discharge Abstract Database
Surgery for renal cell carcinoma	• "Robot-assisted" if 7.SF.14 present (INCODE)<br>• "Laparoscopic" if 1.PC.87.DA OR 1.PD.89.DA or 1.PC.91.DA are present in the absence of 7.SF.14 (INCODE)<br>• Otherwise "Open"	Canadian Institute for Health Information Discharge Abstract Database
Indicative of repair of vena cava and/or thoracoabdominal procedure	Ontario Health Insurance Plan claims with a fee code of E767 or E768 +/- 7 days around date of discharge	Ontario Health Insurance Plan

© 2019 Wettstein MS et al. *JAMA Network Open*.

eTable 2. Qualitative Summary of Subgroup Analyses

Parameter	Subgroup	Radical prostatectomy			Surgery for renal cell carcinoma		
		Before policy change	After policy change	P-value*	Before policy change	After policy change	P-value*
Monthly volume	Overall	↘↘↘	→	0.06	↗↗	↗↗	
	High-volume	↘	→		↗	↗	
	Low-volume	↘↘↘	→		↗↗	↗↗	
Mean length of stay	Overall	↘	↘		↘[a]	↘[a]	
	High-volume	↘	↘		↘[a]	↘[a]	
	Low-volume	↘	↘	< 0.001[b]	↘[a]	↘[a]	
% returning to hospital or ED	Overall	↗	↗		→	→	
	High-volume	↗	↗		↗	↗	
	Low-volume	→	→		→	→	
% older than 65 years	Overall	↗↗	↗↗		→	→	
	High-volume	↗↗	↗↗	0.05[b]	↗↗	↗	
	Low-volume	↗↗	↗↗		→	→	
Mean CCI	Overall	↗↗	→		↘[1]	↘[1]	
	High-volume	↗	→		↗[1]	↘[1]	
	Low-volume	↗↗	→		↘[1]	→[1]	
% minimally invasive approach	Overall	↗↗	↗		↗↗	↗↗	
	High-volume	↗↗↗	↗		↗	→	
	Low-volume	↗↗	→		↗↗	↗↗	

Red in comparison to green background color implies a differential subgroup pattern.
*To improve the readability of the table, p-values greater than 0.1 were not presented.
[a]Visual interpretation impeded by extreme values.
[b]Scientifically irrelevant change.
↗↗↗ | ↗↗ | ↗: strength of upward trend
→: no trend
↘ | ↘↘ | ↘↘↘: strength of downward trend
CCI: Charlson Comorbidity Index; ED: emergency department;

© 2019 Wettstein MS et al. *JAMA Network Open.*

eFigure 1. Derivation of Time Series

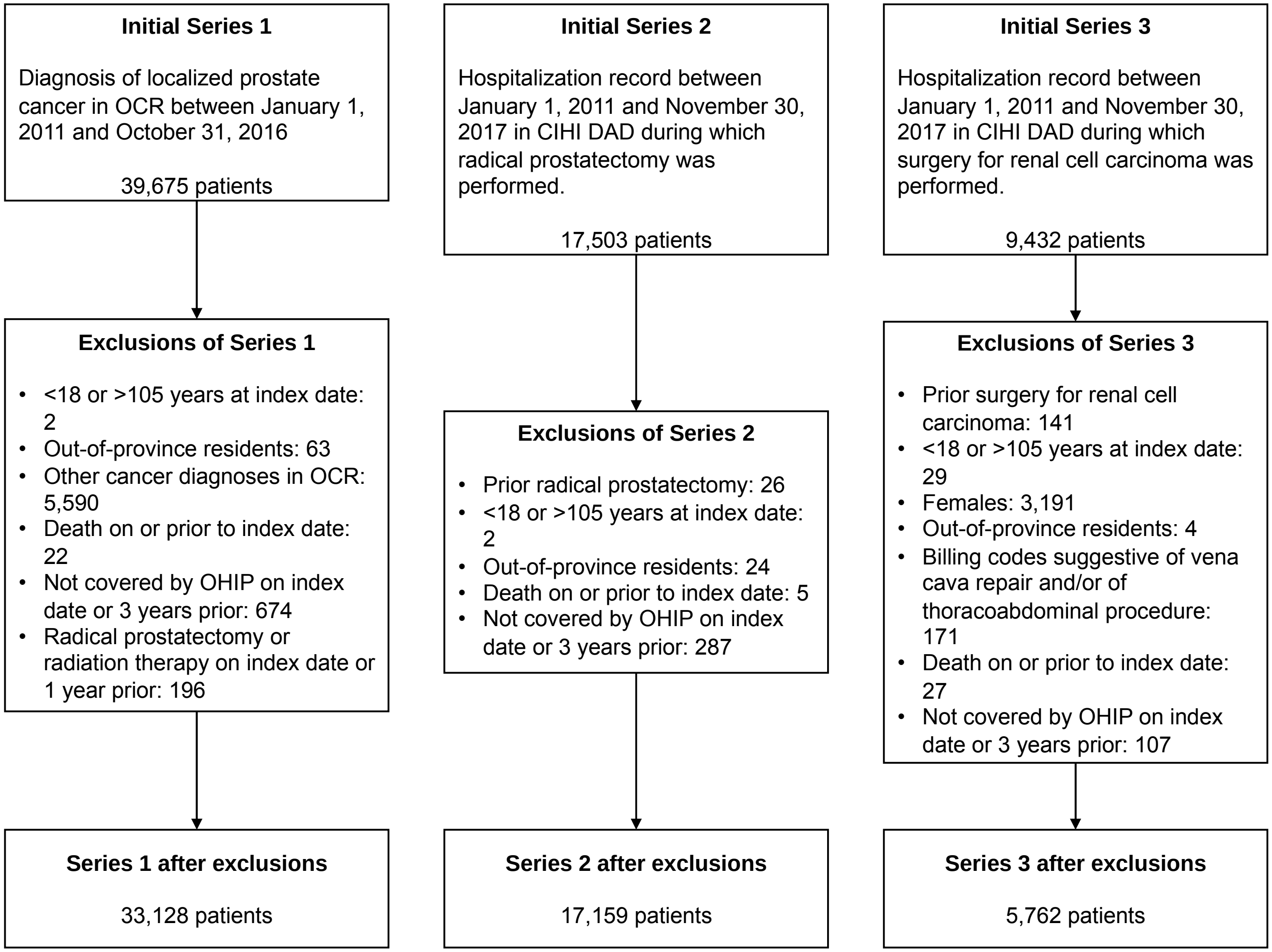

eFigure 1: Derivation of time series. *CIHI DAD: Canadian Institute for Health Information Discharge Abstract Database; OCR: Ontario Cancer Registry; OHIP: Ontario Health Insurance Plan;*

© 2019 Wettstein MS et al. *JAMA Network Open.*

eFigure 2

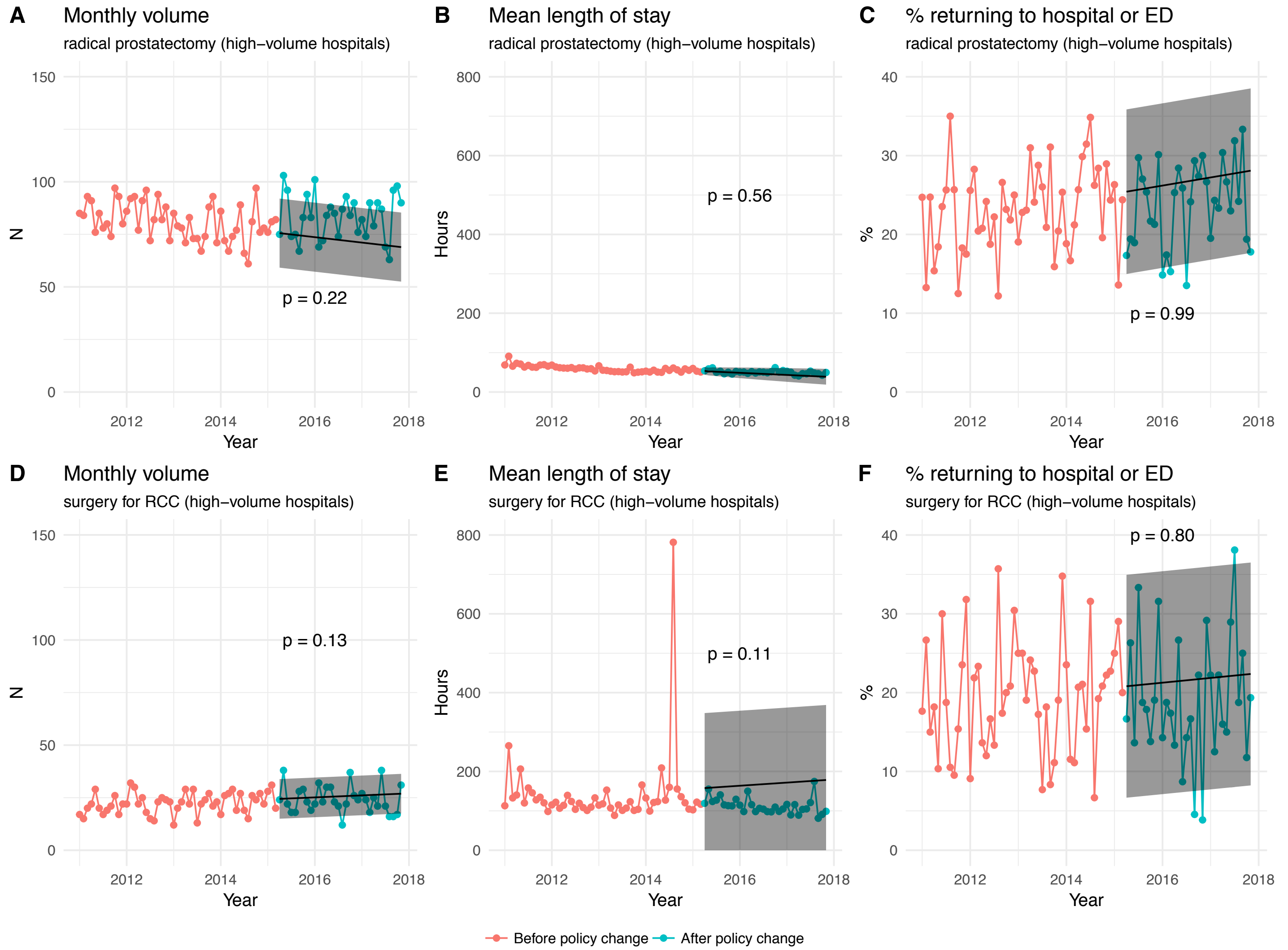

The shaded area represents the predicted 95% confidence interval and the p−value is based on a step function with a zero order response.

eFigure 2: Monthly volume, mean length of stay and proportion of patients returning to the hospital or the emergency department among the subgroup of patients undergoing radical prostatectomy (**A**, **B**, **C**; N: 6,810) and surgery for renal cell carcinoma (**D**, **E**, **F**; N: 1,915) in high-volume hospitals. *ED: emergency department; RCC: renal cell carcinoma;*

© 2019 Wettstein MS et al. *JAMA Network Open*.

eFigure 3

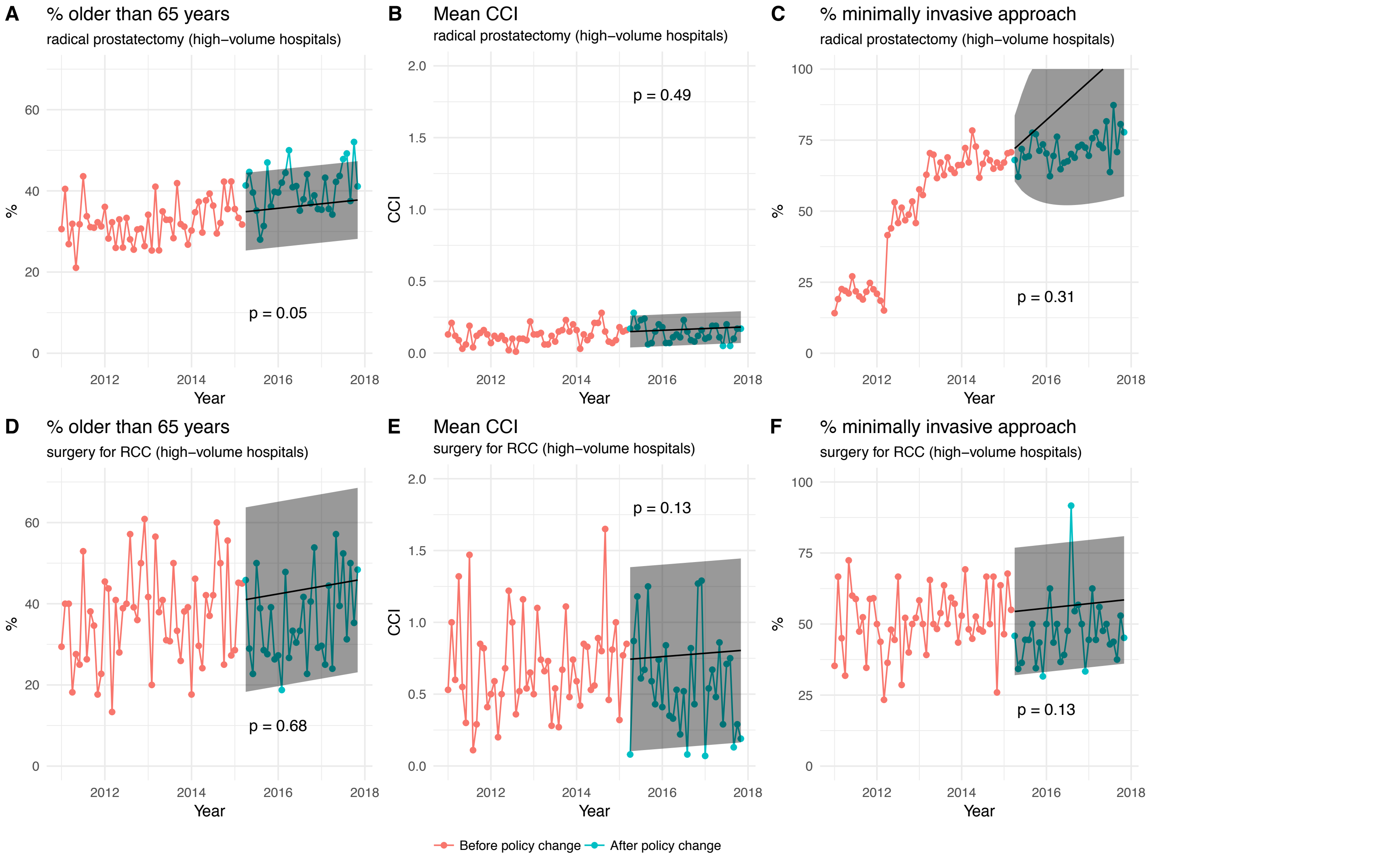

The shaded area represents the predicted 95% confidence interval and the p−value is based on a step function with a zero order response.

eFigure 3: Proportion of patients older than 65 years, mean Charlson Comorbidity Index and proportion of patients who received a minimally invasive approach among the subgroup of patients undergoing radical prostatectomy (**A**, **B**, **C**; N: 6,810) and surgery for renal cell carcinoma (**D**, **E**, **F**; N: 1,915) in high-volume hospitals. *CCI: Charlson Comorbidity Index; RCC: renal cell carcinoma;*

© 2019 Wettstein MS et al. *JAMA Network Open.*

eFigure 4

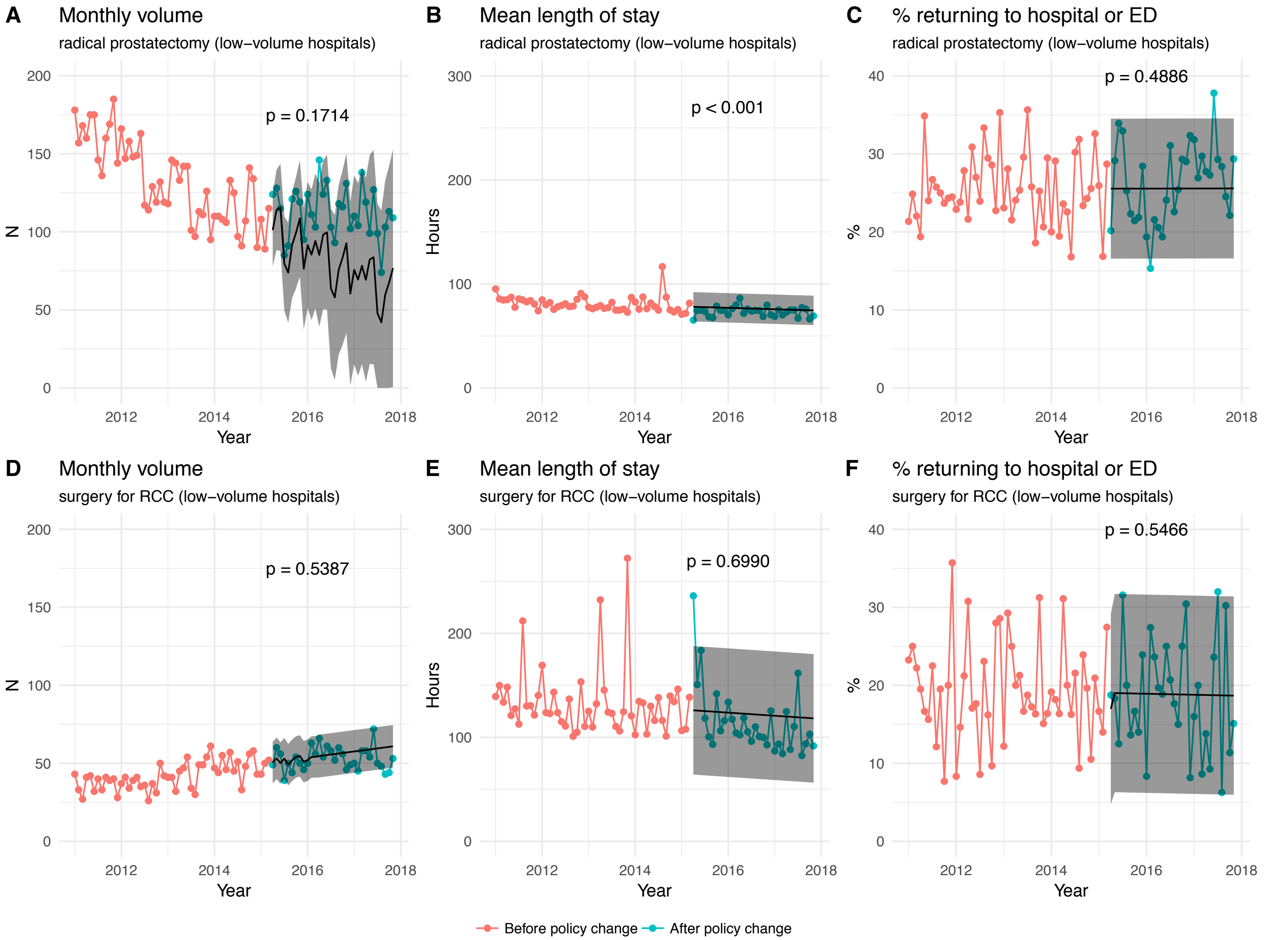

The shaded area represents the predicted 95% confidence interval and the p–value is based on a step function with a zero order response.

eFigure 4: Monthly volume, mean length of stay and proportion of patients returning to the hospital or the emergency department among the subgroup of patients undergoing radical prostatectomy (**A**, **B**, **C**; N: 10,349) and surgery for renal cell carcinoma (**D**, **E**, **F**; N: 3,874) in low-volume hospitals. *ED: emergency department; RCC: renal cell carcinoma;*

© 2019 Wettstein MS et al. *JAMA Network Open*.

eFigure 5

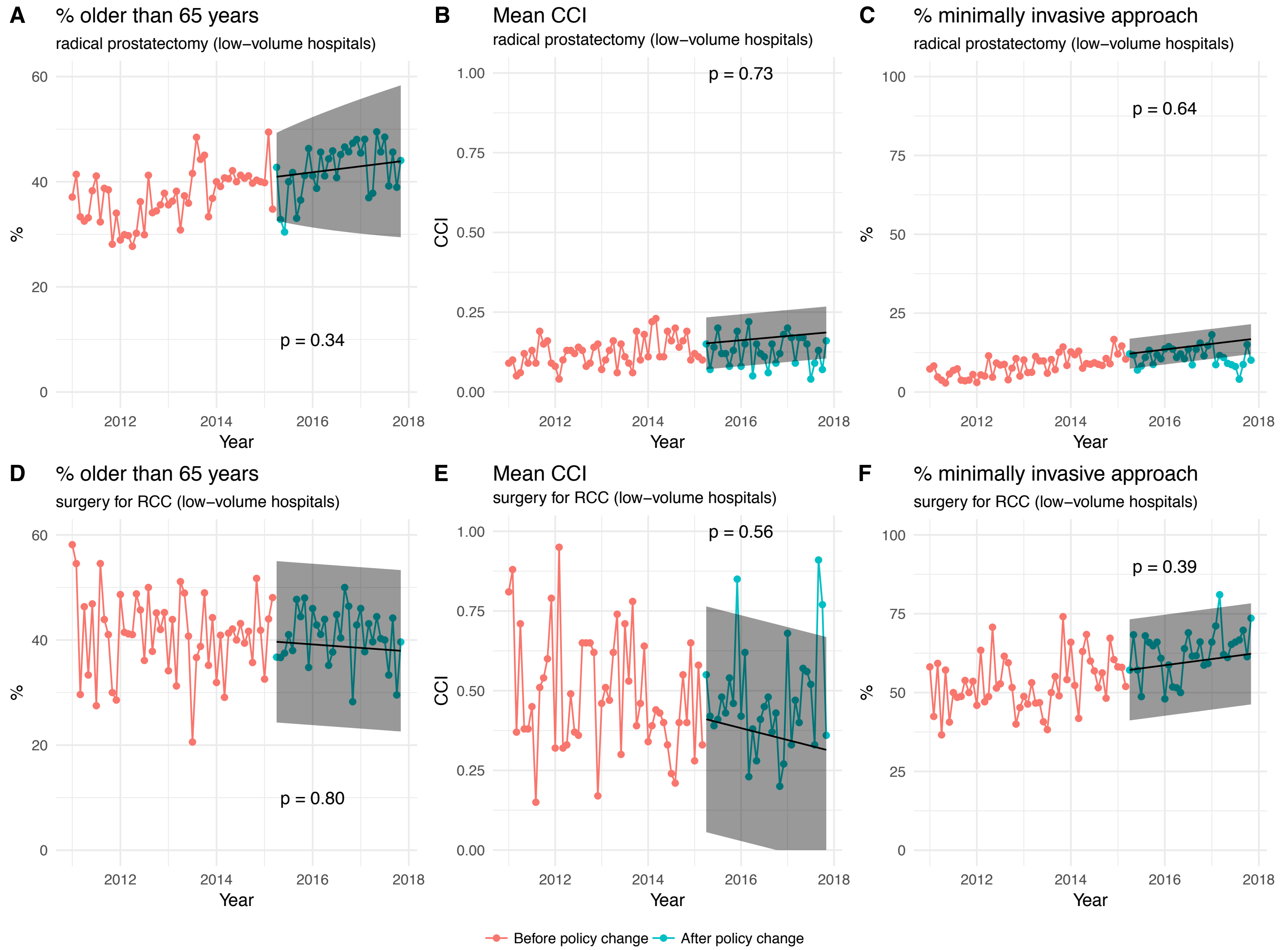

The shaded area represents the predicted 95% confidence interval and the p–value is based on a step function with a zero order response.

eFigure 5: Proportion of patients older than 65 years, mean Charlson Comorbidity Index and proportion of patients who received a minimally invasive approach among the subgroup of patients undergoing radical prostatectomy (**A**, **B**, **C**; N: 10,349) and surgery for renal cell carcinoma (**D**, **E**, **F**; N: 3,847) in low-volume hospitals. *CCI: Charlson Comorbidity Index; RCC: renal cell carcinoma;*

© 2019 Wettstein MS et al. *JAMA Network Open*.

eFigure 6. Step Functions and Model Specifications

(A) Step functions and model specifications of Figure 2

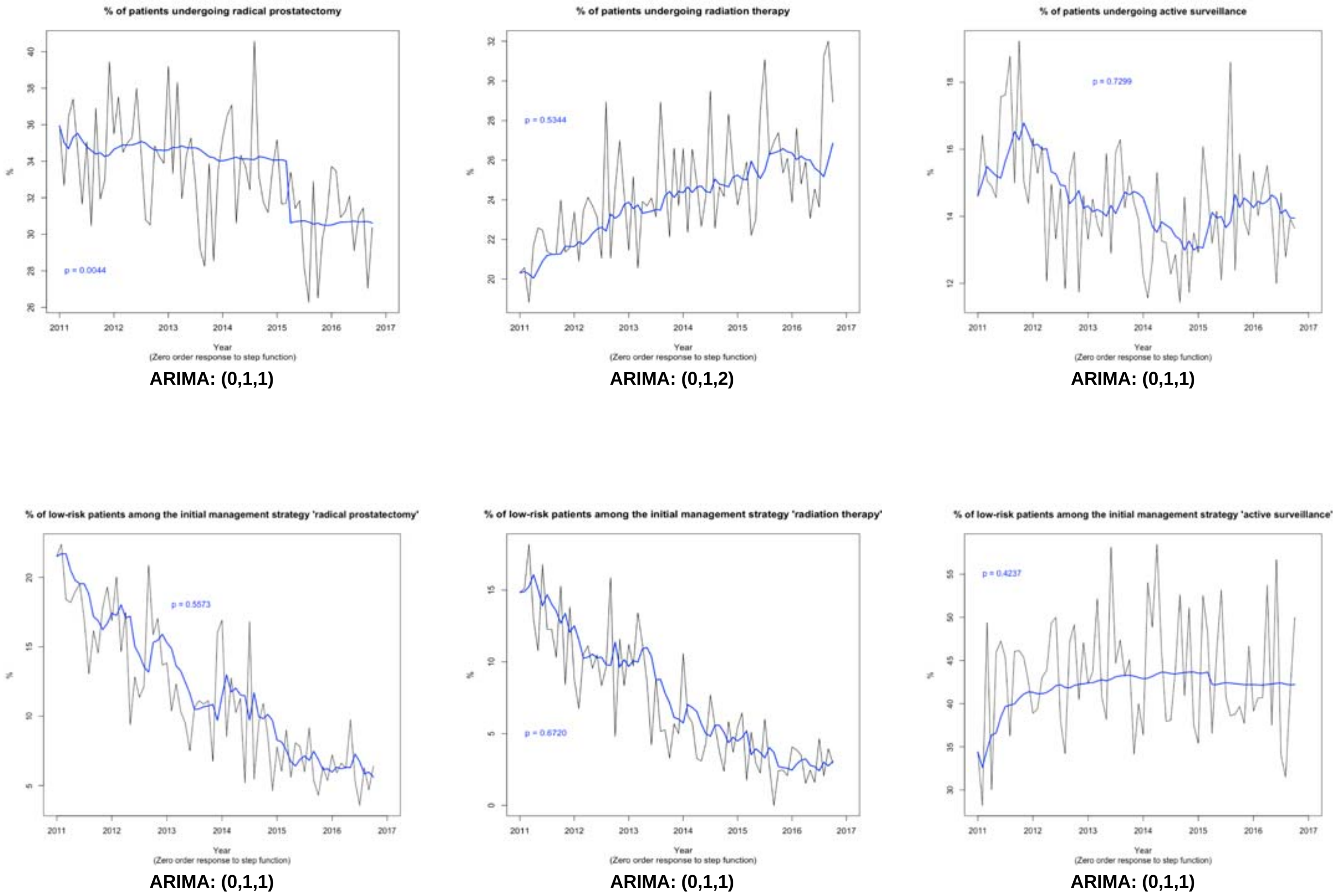

eFigure 6: Step functions and model specifications accompanying *Figure 2* (**A**), *Figure 3* (**B**), Figure 4 (**C**), *eFigure 2* (**D**), *eFigure 3* (**E**), *eFigure 4* (**F**) and *eFigure 5* (**G**).

© 2019 Wettstein MS et al. *JAMA Network Open.*

(B) Step functions and model specifications of Figure 3

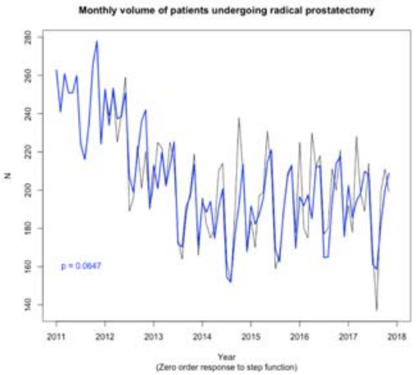

ARIMA: $(0,1,1) \times (0,1,1)_{12}$

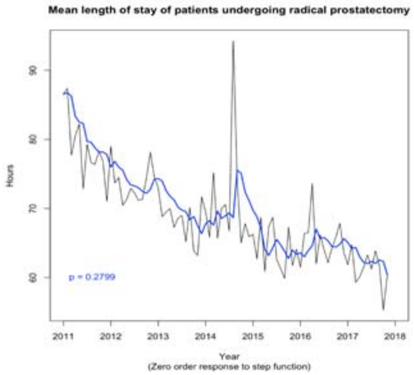

ARIMA: (0,1,1)

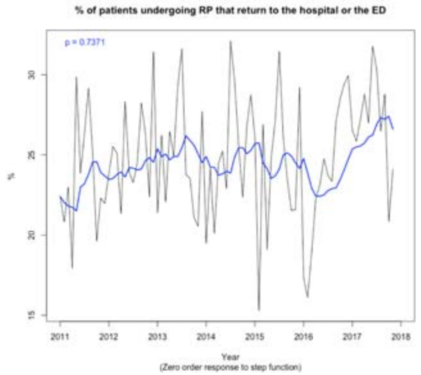

ARIMA: (0,1,1)

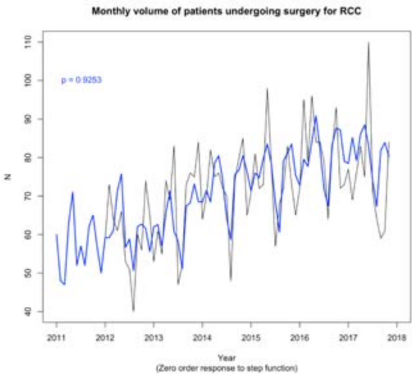

ARIMA: $(0,1,1) \times (0,1,1)_{12}$

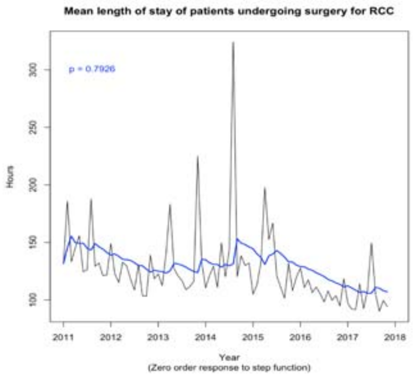

ARIMA: (0,1,1)

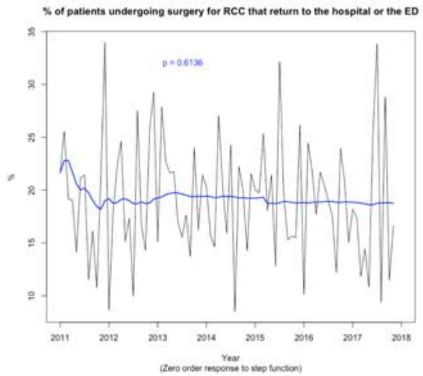

ARIMA: (0,1,1)

eFigure 6: Step functions and model specifications accompanying *Figure 2* (**A**), *Figure 3* (**B**), Figure 4 (**C**), *eFigure 2* (**D**), e*Figure 3* (**E**), *eFigure 4* (**F**) and *eFigure 5* (**G**).

© 2019 Wettstein MS et al. *JAMA Network Open.*

(C) Step functions and model specifications of Figure 4

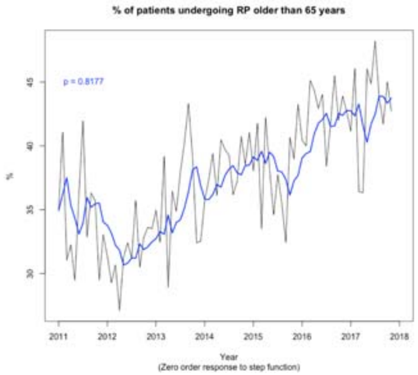

ARIMA: (0,1,1)

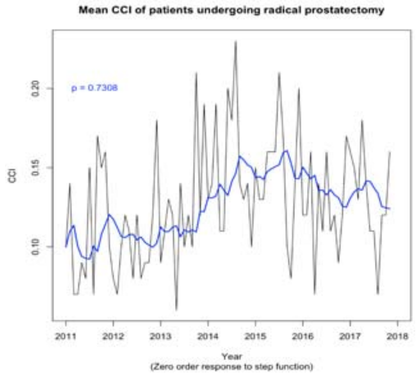

ARIMA: (0,1,1)

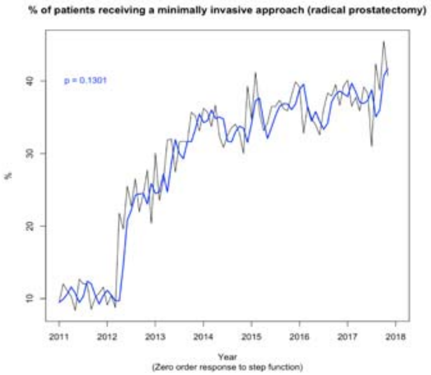

ARIMA: (1,1,0)

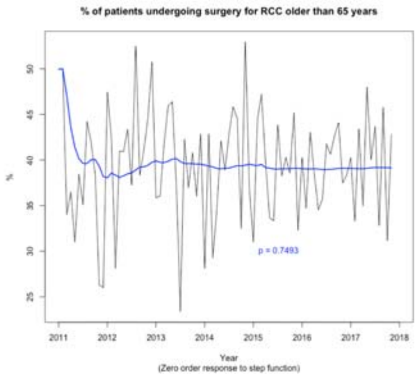

ARIMA: (0,1,1)

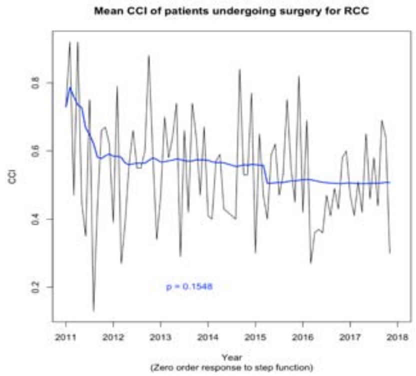

ARIMA: (0,1,1)

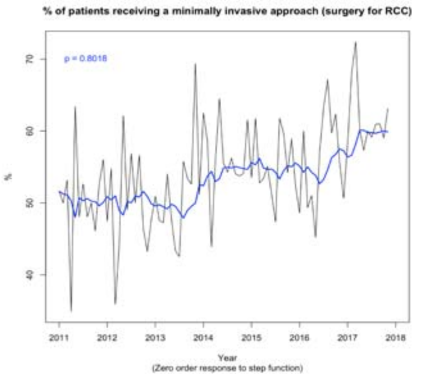

ARIMA: (0,1,1)

eFigure 6: Step functions and model specifications accompanying *Figure 2* (**A**), *Figure 3* (**B**), Figure 4 (**C**), *eFigure 2* (**D**), e*Figure 3* (**E**), *eFigure 4* (**F**) and *eFigure 5* (**G**).

© 2019 Wettstein MS et al. *JAMA Network Open.*

(D) Step functions and model specifications of eFigure 2

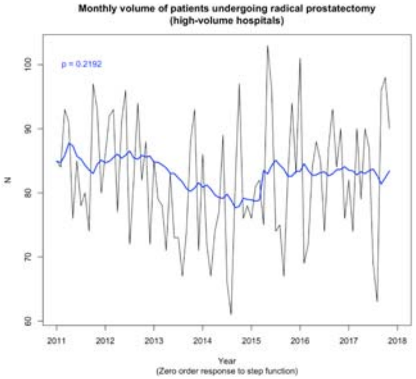

ARIMA: (0,1,1)

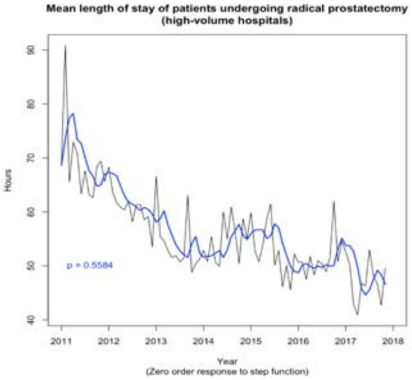

ARIMA: (1,1,1)

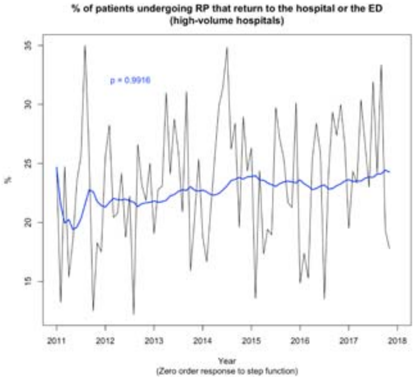

ARIMA: (0,1,1)

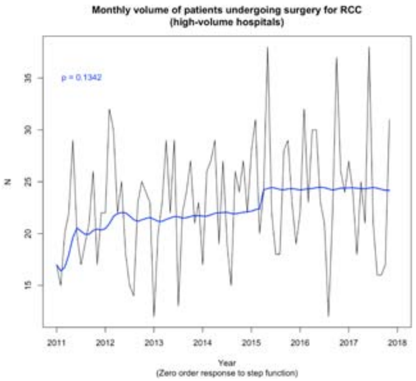

ARIMA: (0,1,1)

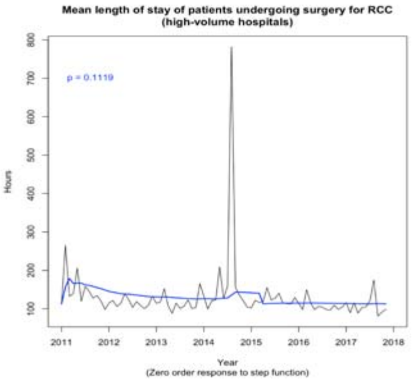

ARIMA: (0,1,1)

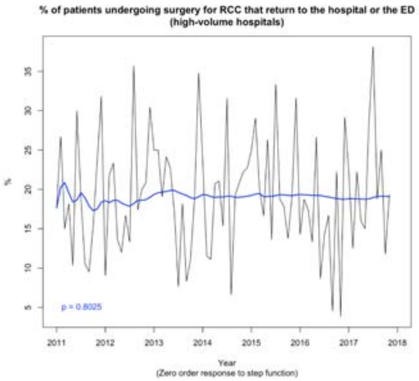

ARIMA: (0,1,1)

eFigure 6: Step functions and model specifications accompanying *Figure 2* (**A**), *Figure 3* (**B**), Figure 4 (**C**), *eFigure 2* (**D**), e*Figure 3* (**E**), *eFigure 4* (**F**) and *eFigure 5* (**G**).

© 2019 Wettstein MS et al. *JAMA Network Open*.

(E) Step functions and model specifications of eFigure 3

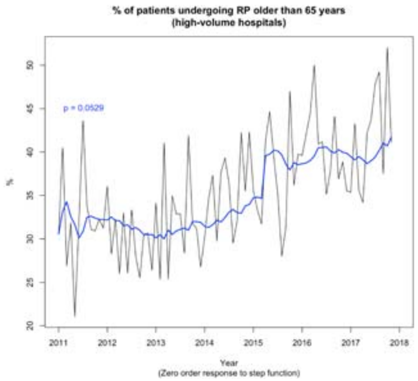

ARIMA: (0,1,1)

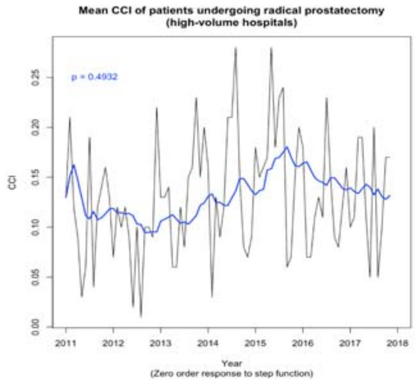

ARIMA: (0,1,1)

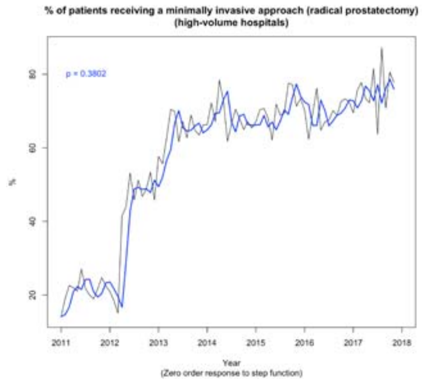

ARIMA: (1,1,0)

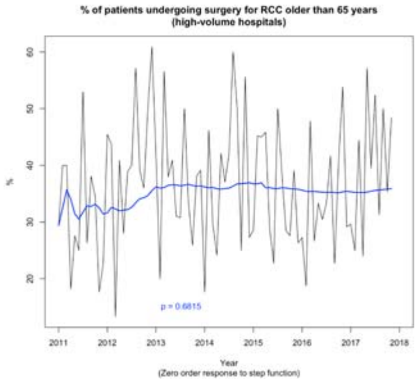

ARIMA: (0,1,1)

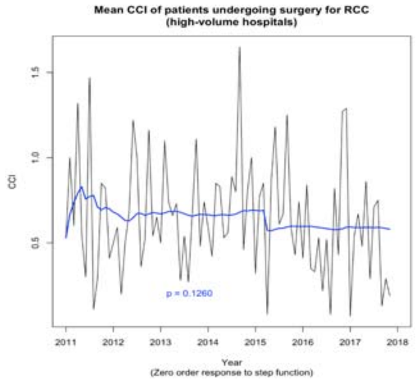

ARIMA: (0,1,1)

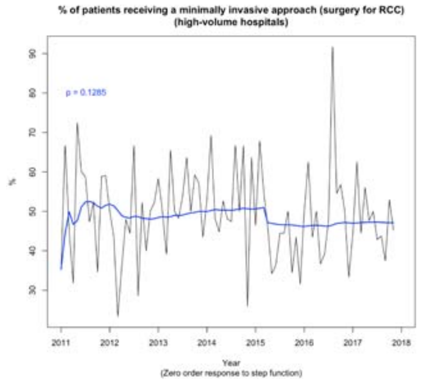

ARIMA: (0,1,1)

eFigure 6: Step functions and model specifications accompanying *Figure 2* (**A**), *Figure 3* (**B**), Figure 4 (**C**), *eFigure 2* (**D**), e*Figure 3* (**E**), *eFigure 4* (**F**) and *eFigure 5* (**G**).

© 2019 Wettstein MS et al. *JAMA Network Open*.

(F) Step functions and model specifications of eFigure 4

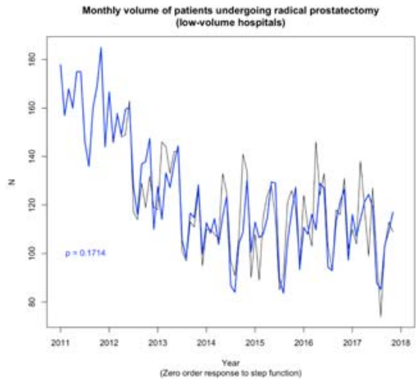

ARIMA: (0,1,1) x $(0,1,1)_{12}$

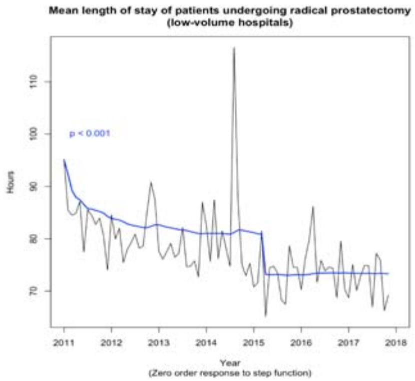

ARIMA: (0,1,1)

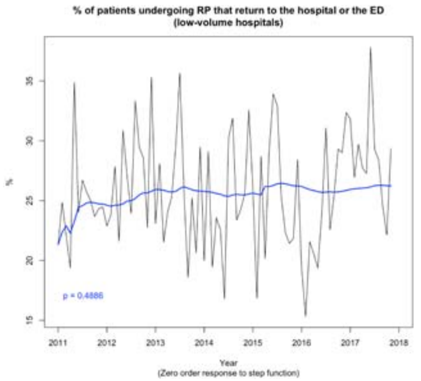

ARIMA: (0,1,1)

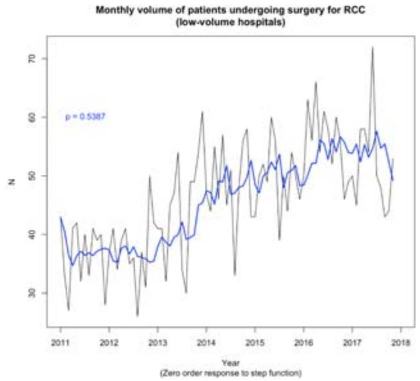

ARIMA: (0,1,1) x $(0,0,1)_{12}$

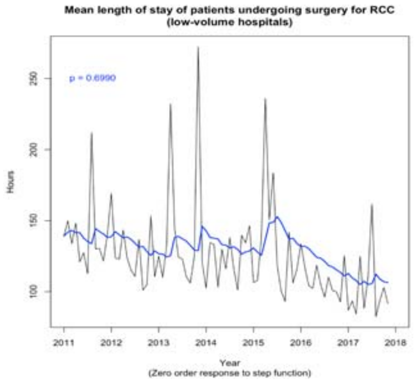

ARIMA: (0,1,1)

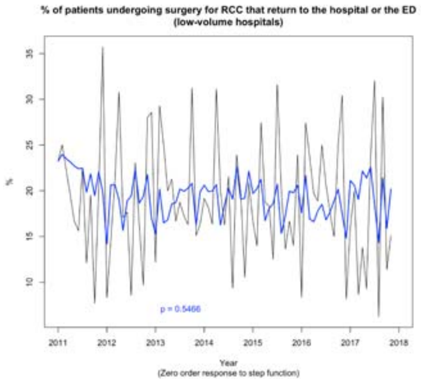

ARIMA: (0,1,2)

eFigure 6: Step functions and model specifications accompanying *Figure 2* (**A**), *Figure 3* (**B**), Figure 4 (**C**), *eFigure 2* (**D**), e*Figure 3* (**E**), *eFigure 4* (**F**) and *eFigure 5* (**G**).

© 2019 Wettstein MS et al. *JAMA Network Open*.

(G) Step functions and model specifications of eFigure 5

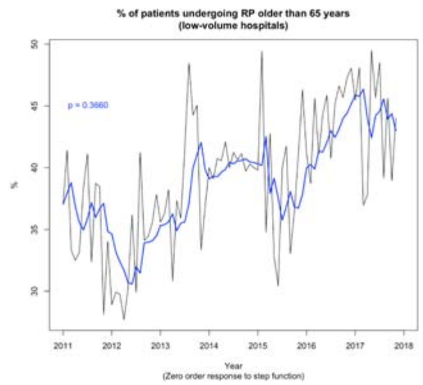

ARIMA: (0,1,1)

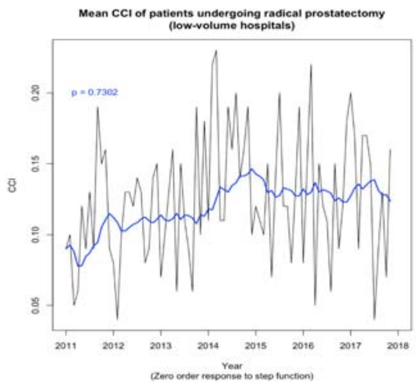

ARIMA: (0,1,1)

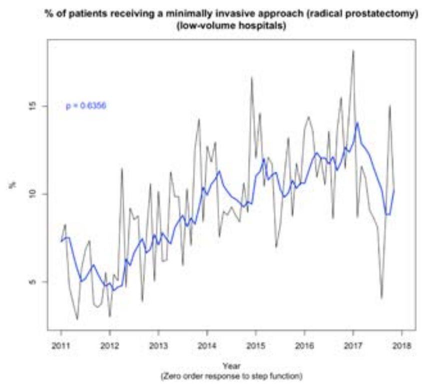

ARIMA: (0,1,1)

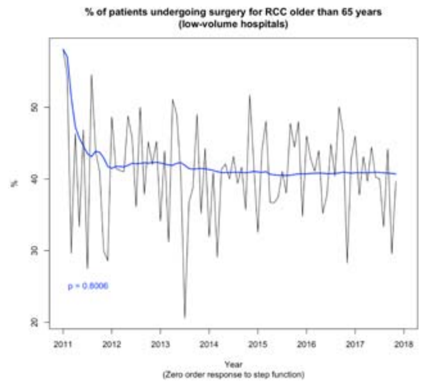

ARIMA: (0,1,1)

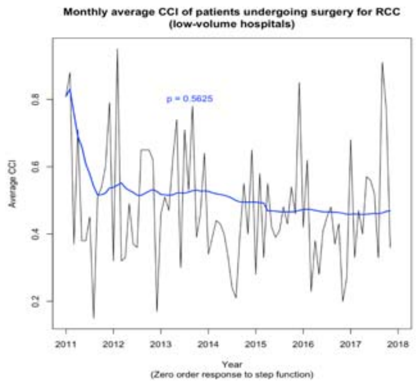

ARIMA: (0,1,1)

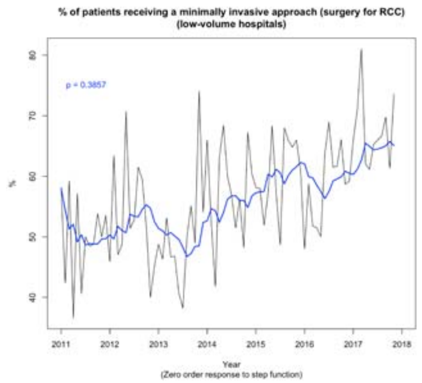

ARIMA: (0,1,1)

eFigure 6: Step functions and model specifications accompanying *Figure 2* (**A**), *Figure 3* (**B**), Figure 4 (**C**), *eFigure 2* (**D**), e*Figure 3* (**E**), *eFigure 4* (**F**) and *eFigure 5* (**G**).

© 2019 Wettstein MS et al. *JAMA Network Open*.
